# How Valid and Reliable Is the International Dysphagia Diet Standardisation Initiative (IDDSI) When Translated into Another Language?

**DOI:** 10.1007/s00455-022-10498-2

**Published:** 2022-08-22

**Authors:** Sara Dahlström, Ida Henning, Jenny McGreevy, Liza Bergström

**Affiliations:** 1 Department of Health and Rehabilitation, Speech and Language Pathology Unit, Institute of Neuroscience and Physiology, Sahlgrenska Academy at the University of Gothenburg, Gothenburg, Sweden; 2 Regional Habilitation Center, Region Kalmar, Oskarshamn, Sweden; 3 Department of Dietetics, Nyköping Hospital, 611 39 Nyköping, Sweden; 4 Centre for Clinical Research Region Sörmland, Eskilstuna, Sweden; 5 Department of Food Studies, Nutrition and Dietetics, Uppsala University, Uppsala, Sweden; 6 Remeo Stockholm, Torsten Levenstams väg 8, SE-128 64 Stockholm, Sweden; 7 Division of Neurology, Department of Clinical Sciences, Karolinska Institutet, Danderyd University Hospital, SE-182 88 Stockholm, Sweden

**Keywords:** Swallowing, Deglutition, Texture-modified consistencies, Dysphagia terminology descriptors

## Abstract

Swallowing difficulties are estimated to affect 590 million people worldwide and the modification of food and fluids is considered the cornerstone of dysphagia management. Contemporary practice uses the International Dysphagia Diet Standardisation Initiative (IDDSI), however, the validity and reliability of IDDSI when translated into another language has not been investigated. This study describes the translation process and confirms the validity and reliability of IDDSI when translated into another language (Swedish). The translation used a 12-step process based on the World Health Organization recommendations. Validity was tested using Content Validity Index (CVI) based on three ratings by a panel of 10–12 experts (Dietitians and Speech-Language Pathologists [SLPs]). The translation was rated for linguistic correlation as well as understandability and applicability in a Swedish context. Inter-rater reliability was calculated using Intraclass Correlation Coefficient (ICC) from 20 SLP assessments of 10 previously published patient cases. Significant improvement (*p* < 0.05) of CVI between Expert Panel assessments was shown for linguistic correlation (improvement from 0.74–0.98) and understandability/applicability (improvement from 0.79–0.93 across ratings). Excellent validity (Item-CVI > 0.78 and Scale-CVI/Average > 0.8) and very high inter-rater reliability (ICC > 0.9) were demonstrated. Results show that, when using a multi-step translation process, a translated version of IDDSI (into Swedish) demonstrates high validity and reliability. This further contributes to the evidence for use of IDDSI.

## Introduction

Dysphagia is prevalent across the life span, from the paediatric to the geriatric populations; it is often complex, multifactorial and a symptom of many congenital, organic, structural, neurological and acquired diseases or syndromes [1–4]. Within the paediatric population, dysphagia may occur as a symptom of prematurity, respiratory and cardiac disorders, gastrointestinal disorders, neurological disorders, congenital anomalies, maternal and perinatal issues, iatrogenic complications and intestinal (caustic) injuries [2]. Dysphagia among adults can occur as a comorbidity in neurodegenerative and age-related neurological diseases [1] such as stroke [5], amyotrophic lateral sclerosis (ALS) [6], multiple sclerosis (MS) [7], Parkinson's disease [8] and Alzheimer's disease [9, 10]. Recent literature also highlights the increasing awareness of presbyphagia and sarcopenic dysphagia in the frail elderly population [11, 12]. Dysphagia in patients with head and neck cancer is significant and debilitating for many patients [13, 14]. In addition, the prevalence of dysphagia in the post-intensive care, post-intubated and tracheostomy populations, including Covid-19 patients, has been increasingly reported in the literature during recent years [15–17].

The consequences of dysphagia are many, varied and may be serious or life-threatening in nature, including aspiration pneumonia, malnutrition, dehydration, with accompanying diminished health and quality of life [18–22]. Furthermore, dysphagia is an independent factor contributing to increased hospital lengths of stay and healthcare costs [15, 19, 23, 24].

Given the impact dysphagia has on a person’s health and quality of life, and the societal costs, the importance of optimal dysphagia management has been underscored by several research groups [4, 15, 25, 26]. Dysphagia is often managed with compensatory and/or rehabilitative strategies or exercises [4, 26, 27]. In terms of compensatory strategies, texture-modified consistencies (TMC) of food and drink are considered the cornerstone of management [3, 26, 28].

Although TMC are used throughout the world in everyday dysphagia management, the range of modified food and fluid consistencies, the variety of names, the number of different levels of modification and the characteristics used to describe TMC are many and varied [29–32]. Such variability and non-standardisation within and across hospitals, healthcare regions, and countries negatively impact dysphagia management and patient safety [29–32]. In Sweden, for example, variability in the terms used to describe texture modified consistencies is known to exist between different regions, hospitals and also within the same hospital and/or region, with over 70 different TMC terms used by SLPs across the country [32]. This is reported to negatively impact optimal multidisciplinary teamwork, communication and dysphagia management, and increase patient safety risks [29, 32].

The importance of TMC terminology and the correct understanding of descriptors used by healthcare professionals to communicate TMC continue to be highlighted in dysphagia literature. In 2015, an international multidisciplinary group created the International Dysphagia Diet Standardisation Initiative (IDDSI) in an attempt to address these shortcomings. IDDSI provides standardised terms, descriptors and measurement criteria for TMC food and drinks and has been developed to be used worldwide across all cultures [29, 33]. IDDSI implementation internationally continues to grow [33] and IDDSI has, to date, been translated into 19 languages with another 12 languages currently in translation process.

At the time of manuscript preparation, variability in dysphagia terminology used by SLPs and other health professions throughout Sweden continues to be prevalent. Subsequently, this research group undertook the translation and cultural adaptation of IDDSI into Swedish. Previous research has demonstrated good validity and reliability of the IDDSI framework in English [34], however, to the best of the authors’ knowledge, this is the first study to investigate the validity and reliability of IDDSI when translated into another language. Using a multi-step translation method, based on the WHO guidelines, the following research questions were investigated:

1. Does the Swedish translation of IDDSI show high content validity regarding linguistic correlation and understandability/applicability in a Swedish context?
2. Is the inter-rater reliability for the Swedish translation of IDDSI of a high level?

## Method

There is no standardised method for the translation of material from one language to another [31, 35]. The World Health Organization's guidelines [36] use a four-step process to produce a conceptually equivalent, rather than a literal, translation. The method used in the present study was based on the World Health Organization’s guidelines, however, enhanced to an improved multi-step translation process [31, 37].

### Procedure

**Fig. 1:**
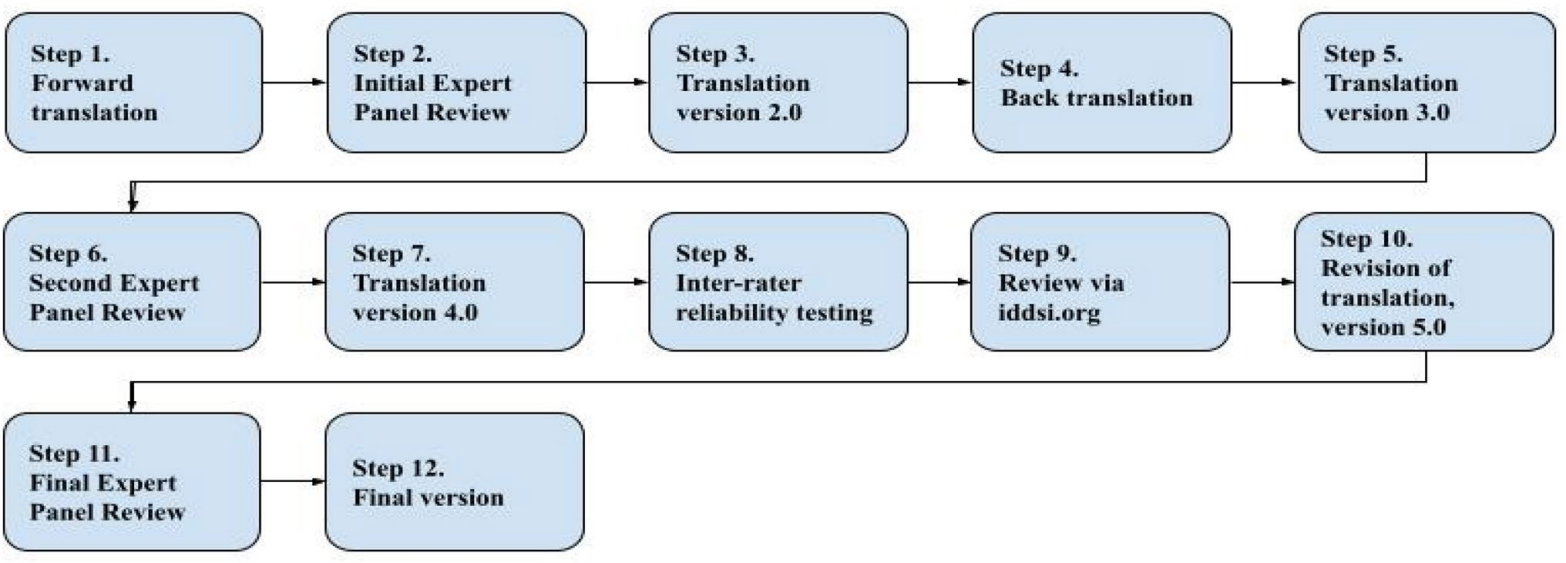
Flowchart of 12-step translation process

The 12-step translation and cultural adaptation process used forward and back translation, and expert reviews, as follows: (1) Forward translation; (2) Initial Expert Panel Review; (3) Translation version 2.0; (4) Back translation; (5) Translation version 3.0; (6) Second Expert Panel Review; (7) Translation version 4.0; (8) Inter-rater reliability testing; (9) Review via iddsi.org.; (10) Revision of translation, version 5.0; (11) Final Expert Panel Review; and (12) Final version (see Fig. 1).

### Translation

The forward translation of the IDDSI framework and testing methods (Step 1) was performed by the authors: one SLP, one dietitian and two SLP Magister students (first year of a master’s degree). All authors have in-depth understanding of the IDDSI concepts. Two are native English speakers who have lived in Sweden for 10–28 years and two are native Swedish speakers; all are proficient in both languages. Regular discussions were held, and consensus decisions made in the four areas of equivalence [38]; semantic (words mean the same, no double meanings); idiomatic (idioms hard to translate); experiential (daily life experiences); and conceptual (concepts used may differ across cultures and languages). Linguistic and cultural aspects were, therefore, considered during the process to ensure the translation fully reflected the Swedish context. For example, timbal, an established concept in Sweden made from puréed foods mixed with egg and baked, was added to IDDSI level 4.

This translation was then sent to the Expert Panel (Step 2) for their initial review and rating of Translation version 1 (Expert Panel further described below). Following review and feedback, the IDDSI translation was adapted to create Translation version 2 (Step 3) prior to the back translation.

Back translation of the material from Swedish to English was performed in Step 4 by two translators separately: both were naive to the IDDSI material and context, as per WHO guidelines. The back-translators (one male, one female, age range 32–47 years) were recruited through author contacts. One translator was bilingual, the second had English as their native language with advanced level of Swedish as per the Common European Framework of Reference (CEFR). The back-translators also provided comments that were then taken into account during Step 5 (the revision and production of Translation version 3).

### Expert Panel

The 12 participants in the Expert Panel consisted of four speech-language pathologists (SLPs) and eight dietitians; 10 female, 2 male; age range 29–60 + years. All participants had at least 3 years of clinical experience working in the area of dysphagia management and good knowledge of Swedish and English. Expert Panel participants were recruited through the Swedish National Dysphagia Network as well as with use of the snowball method [39], whereby participants could forward recruitment details to other potentially interested professionals. Recruitment aimed to ensure maximum possible variation regarding age, geographical location and number of years of experience working with dysphagia management. The same Expert Panel was used in Steps 2, 6 and 11, where each new translation was reviewed and rated. Two Expert Panel members (1 × SLP/1 × dietitian) were not able to participate in the final, third review, however, this was not considered to be detrimental to the review process since a panel ≥ 10 reduces the risk of “chance” agreement [40].

### Validity

The Expert Panel reviewed and rated the translation of each IDDSI level with respect to (a) linguistic correlation and (b) understandability and applicability in a Swedish context. Each item was rated on the following four-point scale: 1 = no correlation, not understandable/applicable; 2 = slight correlation, slightly understandable/applicable; 3 = quite good correlation, quite understandable/applicable; 4 = very good correlation, very understandable/applicable. During their review, the Expert Panel members were also encouraged to provide suggestions for improving the translation.

The validity of the translation was calculated using (a) Item-Content Validity Index (Item-CVI), where the term “item” is used to describe the different IDDSI levels, and (b) Scale-Content Validity Index (Scale-CVI/Average), which is the average (mean) Item-CVI for all items [40]. The CVI results and Expert Panel suggestions were taken into consideration to produce the next version of the IDDSI translation in Steps 3, 7 and 12.

### Inter-rater Reliability

**Fig. 2:**
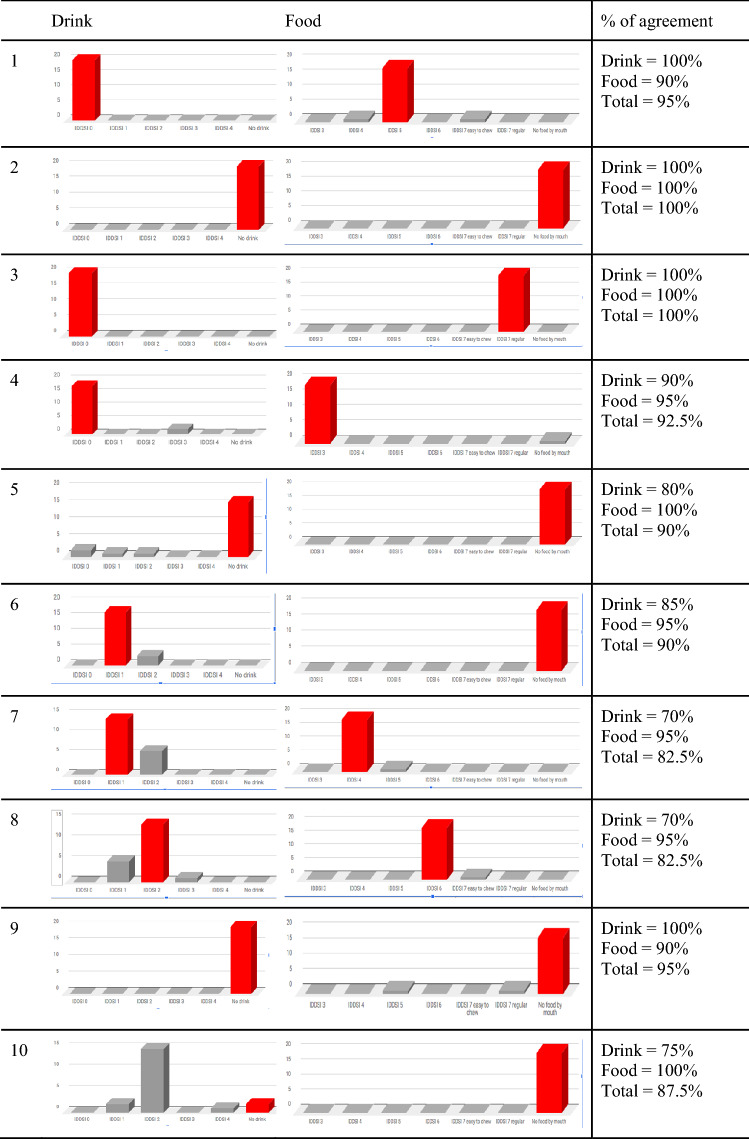
Results of patient cases reported separately divided into food and drink. Posts marked in red show the results of the patient cases as reported by Steele et al., 2018

Inter-rater reliability (see Fig. 2) was calculated with 20 Swedish SLPs (16 female, 3 male, 1 information not provided) using IDDSI Translation version 4.0, where each SLP provided consistency recommendations for 10 fictitious patient cases, previously published within IDDSI literature [34]. Recruitment occurred through Swedish SLP Facebook groups with the following inclusion criteria: registered SLP in Sweden, working with dysphagia, and able to read and understand academic English. Recruited SLPs were between 29 and 60 + years of age. The participants worked in different areas of speech pathology, including habilitation, general speech therapy, medical, neuro and within Ear–Nose–Throat (ENT) caseloads. Recruitment aimed to obtain a large spread in age, regional affiliation, workplace and years of experience.

### Final Review (as per iddsi.org Website)

Following Step 9, and in accordance with IDDSI [33] regulations, Translation version 4.0 was made available on www.iddsi.org for three months from May 2020, providing opportunity for public comments and suggestions. Based on this feedback, the authors produced Translation version 5.0, which underwent a final Expert Panel review (Steps 10 and 11) before establishing the final version that was published on iddsi.org in January 2021.

## Statistical Analysis

Descriptive statistics were calculated, and inferential statistical analysis performed using the IBM SPSS Statistics program, version 25.

The CVI method was used to measure inter-rater agreement for the translation of each IDDSI level (Item-CVI) using the ratings by the Expert Panel shown in Table 1. The ratings were collapsed into a dichotomy (ratings 1 & 2 versus 3 & 4) where the Item-CVI is the proportion of experts giving a positive/acceptable rating (3 or 4) for each item. The minimum recommended Item-CVI value is 0.78, indicating good content validity [40]. Scale-CVI/Average calculates the average Item-CVI for all items and 0.90 is the cut-off indicating good content validity [40]. The difference between the ratings in the initial, second and third (final) Expert Panel Reviews (ordinal data) was also calculated using Wilcoxon signed-rank test. Significance level *p* < 0.05 was chosen. It should be noted that differences in calculation methods for (a) the Item-CVI (ratings collapsed into a dichotomy) and (b) the Wilcoxon signed-rank test may produce results which are not comparable statistically, a reflection of their different calculation methods.

**Table 1:** Linguistic ratings of IDDSI translation into Swedish according to the expert panel using the Item-Content Validity Index (CVI) and scale-CVI/average

	Item-CVI	Item-CVI	Item-CVI	Difference in Item-CVI from expert panel ratings	
	Initial expert panel review *n* = 12	Second expert panel review *n* = 12	Third expert panel review *n* = 10	1–2	2–3
IDDSI 0	1	–	–		
IDDSI 1	0.58	1	–	0.009*	
IDDSI 2	0.92	1	–	0.025*	
IDDSI 3	0.75	1	–	0.02*	
IDDSI 4	0.67	1	–	0.01*	
IDDSI 5	0.5	0.83	0.9	0.238	0.785
IDDSI 6	0.5	1	–	0.016*	
IDDSI 7 easy to chew	0.83	1	–	0.053	
IDDSI 7 regular	0.92	1	–	0.157	
Transitional foods	0.67	1	–	0.011*	
Scale-CVI/average	0.74	0.98	(n.c.)		
Difference between expert panel ratings 1–2 and 2–3				< 0.001	(n.c.)

High values indicated by Item-CVI > 0.78, and Scale-CVI/Average > 0.80. Significance with *p* < 0.05. Difference between the initial and second expert panel review ratings was calculated using Wilcoxon signed-rank test based on the raw (ordinal) data not the Item-CVI collapsed dichotomy data. Scale-CVI/Average was not calculated (n.c.) for the third expert panel review ratings since only one item was rated. Similarly, difference in the second and third expert panel review ratings was also not calculated (n.c.)

Inter-rater reliability was calculated using the Intraclass Correlation Coefficient (ICC) with a confidence interval of 95%. Calculation of ICC occurred using a “two-way mixed” model with “absolute agreement”. The value “average measures” was used to compare the participants’ assessments. “Single measures” were also calculated to measure the reliability of a typical participant. An ICC value ​​of < 0.5 indicates poor reliability, 0.5–0.75 moderate reliability, 0.75–0.9 good reliability and > 0.9 indicates very good reliability [41]. Cronbach’s alpha, used to calculate internal consistency, measured the extent to which the variables correlated with each other, where a value > 0.7 indicates good internal reliability [42].

## Results

### Validity of the Swedish Translation of IDDSI: Linguistic Correlation

For linguistic correlation, results from the initial Expert Panel Review for Item-CVI showed that 5/11 items demonstrated an Item-CVI > 0.78, indicating good content validity [40]—see Table 1. An improvement in Item-CVI was demonstrated after the second Expert Panel Review, with all 11 items meeting requirements for excellent content validity with most improvements being statistically significant (*p* < 0.05). A few small spelling mistakes were highlighted and corrected in step 9: review via iddsi.org. Only one item (IDDSI 5) was rated for linguistic correlation a third time by the Expert Panel, since all other items demonstrated excellent content validity (1.0). The Item-CVI of this item (IDDSI 5), already demonstrating good validity > 0.78), improved non-significantly to 0.9. The Scale-CVI/Average increased from 0.74 to 0.98, demonstrating excellent overall scale validity for linguistic correlation [40].

### Validity of the Swedish Translation of IDDSI: Understandability/Applicability in a Swedish Context

Regarding the IDDSI translation being understandable and applicable in a Swedish context, the results from the initial Expert Panel Review showed that 5/11 items demonstrated good validity (Item-CVI > 0.78)—see Table 2. From the ratings of the second Expert Panel Review, all 11 items met the requirements for good validity with 5/11 demonstrating a perfect Item-CVI of 1. From the third Expert Rating Review, all Item-CVI values were high = 0.9, therefore no statistically significant improvement occurred between the second and third Expert Panel Reviews.

**Table 2:** Understandability and applicability ratings of IDDSI translation into Swedish according to the expert panel using the Item-Content Validity Index (CVI) and scale-CVI/average

	Item-CVI	Item-CVI	Item-CVI	Difference in Item-CVI from Expert Panel ratings	
	Initial Expert Panel review *n* = 12	Second Expert Panel review *n* = 12	Third Expert Panel review *n* = 10	1–2	2–3
IDDSI 0	1			0.705	
IDDSI 1	0.67	1		0.058	
IDDSI 2	0.92	1		0.48	
IDDSI 3	0.75	1		0.166	
IDDSI 4	0.75	0.83	0.9	0.034*	0.564
IDDSI 5	0.67	0.83	0.9	0.565	0.739
IDDSI 6	0.67	1		0.07	
IDDSI 7 easy to chew	0.92	0.92	0.9	0.739	0.317
IDDSI 7 regular	0.83	0.83	0.9	0.608	1
Transitional foods	0.67	0.92	0.9	0.096	0.083
Scale-CVI/Average	0.79	0.93	0.9		
Difference between expert panel ratings 1–2 and 2–3				0.02*	0.32

High values indicated by Item-CVI > 0.78, and Scale-CVI/Average > 0.80 and significance with *p* < 0.05. Difference between initial and second expert panel review ratings and second and third expert panel review ratings were calculated using Wilcoxon signed-rank test based on the raw (ordinal) data not the Item-CVI collapsed dichotomy data

### Inter-rater Reliability

Results demonstrated very high inter-rater reliability (ICC > 0.9) with a 95% confidence interval for both food and drink (see Table 3). For detailed rater agreement for each patient case, see Fig. 2.

**Table 3:** Inter-rater reliability, calculated via Intra Class Correlation (ICC)

	Food + Drink	Food	Drink
Cronbach’s Alpha	0.995	0.997	0.996
Average measures	0.995	0.997	0.997
Single measures	0.908	0.942	0.937
*p* value	< 0.001*	< 0.001*	< 0.001*

*Significance where *p* < 0.05; Cronbach’s Alpha > 0.7; Average measures > 0.9; Single measures > 0.9

## Discussion

The purpose of this study was to assess the validity and reliability of IDDSI when translated into another language (Swedish) using strong methodology with a multi-step translation process. Excellent results for Content Validity (Item-CVI > 0.78 and Scale-CVI/Average > 0.90) were achieved regarding (a) how well the Swedish IDDSI translation correlated *linguistically* with the English version and (b) if the Swedish IDDSI translation was considered *understandable and applicable* in a Swedish context [40]. The reliability results for the 10 fictitious patient cases (as per previous IDDSI research) showed very high inter-rater reliability (ICC > 0.9) using the Swedish-translated IDDSI.

### Validity of Swedish Translation of IDDSI

Calculation of CVI provides quantified evidence that the translated items and scale are content valid. The Scale-CVI improved significantly (*p* ≤ 0.02) across the Expert Panel reviews in both (a) linguistic correlation and (b) understandability and applicability in a Swedish context—particularly from Expert Panel 1 to Expert Panel 2. It is interesting to note that, although excellent results were achieved for Item-CVI after Expert Panel 2, there were slight differences regarding the results for *linguistic* correlation as compared to *understandability and applicability* in a Swedish context. For example, for *linguistic* correlation, higher results were achieved earlier, as compared with the *understandability and acceptability* correlation and CVI. This highlights the importance of (1) testing the content validity of a translated scale considering both linguistic and cultural considerations, and (2) using a multi-step process, as reported by earlier studies [30, 35, 38, 42].

The current study used an Expert Panel on three occasions with revision and improvements incorporated after each review. Such steps are considered essential, particularly, for the linguistic correlation as reported by previous research [40, 41], where the importance of the Expert Panel's input for adapting language and word choice in a translation is highlighted. Similarly, the current study also demonstrated improved versions both linguistically and regarding understandability/applicability following a review of comments and suggestions from the Expert Panel. Additionally, the varied Expert Panel with *n* ≥ 10 is also considered an important aspect contributing to the high-level translation outcome since the risk of chance agreement diminishes with an increased number of experts on the panel [40].

### Rater Reliability Using IDDSI (Swedish Translation)

Since the 20 SLPs who participated in the assessment of patient cases were of varying ages, range of experience, and from different clinical areas and regions in Sweden, the high inter-rater reliability (ICC = 0.99) is considered to be generalisable to the Swedish SLP population (*p* < 0.001). Interestingly, for patient case 10, the Swedish SLPs demonstrated high reliability but gave an ‘incorrect’ assessment according to the original reference [34]. The patient case concerns a middle-aged man who, due to cancer, received his main nutrition via gastrostomy. He silently aspirated with IDDSI 0 and IDDSI 1. He had been advised to continue using the gastrostomy but to try to swallow small amounts of IDDSI 2 to maintain normal swallowing. The reference materials recommend “no drink” but 15 out of 20 Swedish SLPs suggested IDDSI 2 as the answer, which was the consistency the man was recommended to practice swallowing with. Thus, 15 out of 20 SLP were in agreement but gave an ‘incorrect’ recommendation (compared with the results), suggesting that the patient case was unclear and therefore open to different interpretations. This disparity may be reflective of different medico-legal influences and international practices. Future research should investigate both intra and inter-rater reliability perhaps using real patient assessments via recorded FUS / VFSS.

## Limitations and Future Research

As with all research, the current study has limitations. In terms of inter-rater reliability, this is an area for future improvement. The patient cases used were prepared by the Dysphagia Diet Standardisation Committee for the purpose of validating original IDDSI material [34] and similarly, were presented in English, even in the present study. Although all SLPs were Swedish and were required to have proficient English (inclusion criteria), it is difficult to know if the reliability results were affected by these English-written cases. In the validation of the original material, SLPs from all over the world responded [34] and few of these SLP were reported to have had English as their first language. Despite this limitation, the reliability was still very high (ICC = 0.99). Furthermore, the inter-rater reliability, although very high, was calculated using IDDSI Translation version 4.0 prior to uploading to idds.org website and final feedback. Future research should evaluate both inter-rater and intra-rater reliability using the final IDDSI version. Finally, IDDSI has been described [29] to be a living document that needs to be kept up to date using the developments from research. To further strengthen the validation of the Swedish translation of the IDDSI material, future research should investigate whether patients and their relatives are able to understand and prepare texture modified food and drink according to IDDSI.

## Conclusion

To the authors knowledge this is the first study investigating the validity and reliability of IDDSI when translated into another language (Swedish). The multi-step translation process resulted in excellent validity and very high inter-rater reliability, which paves the way for implementation of IDDSI in Sweden. The use of the standardised terminology, descriptors and testing methods for texture modified consistencies is expected to improve communication between professions, patients and relatives, which in turn, is expected to lead to increased patient safety and dysphagia management.
